# Curcumin-Loaded Oil-Free Self-Assembled Micelles Inhibit the Influenza A Virus Activity and the Solidification of Curcumin-Loaded Micelles for Pharmaceutical Applications

**DOI:** 10.3390/pharmaceutics14112422

**Published:** 2022-11-09

**Authors:** Cun-Zhao Li, Hui-Min Chang, Wei-Li Hsu, Parthiban Venkatesan, Martin Hsiu-Chu Lin, Ping-Shan Lai

**Affiliations:** 1 Department of Chemistry, National Chung Hsing University, No. 145, Xingda Road, Taichung 402, Taiwan; 2 Graduate Institute of Microbiology and Public Health, National Chung Hsing University, No. 145, Xingda Road, Taichung 402, Taiwan; 3 Ph.D. Program in Tissue Engineering and Regenerative Medicine, National Chung Hsing University, No. 145, Xingda Road, Taichung 402, Taiwan; 4 Department of Neurosurgery, Chang Gung Memorial Hospital, Chia-Yi Branch, Chia-Yi 613, Taiwan

**Keywords:** curcumin-loaded micelles, influenza A virus, self-assembly, solidification, pharmaceutical formulations

## Abstract

Curcumin, a well-known natural lipophilic phenolic compound, plays a vital role in inhibiting the influenza infection. Currently, many kinds of formulations for the enhancement of a water dispersion of curcumin have been developed; however, the anti-influenza abilities of formulated curcumin have been much less investigated. In this study, the optimized self-assembled micelles of RH 40/Tween 80 loaded with curcumin (Cur-M) in an oil-free-based system were spherical with a hydrodynamic size at 13.55 nm ± 0.208 and polydispersity at 0.144 characterized by atomic force microscopy and dynamic light scattering, respectively. Additionally, Cur-M significantly increased the bioactivity/stability of curcumin and effectively inhibited the influenza A virus infection and its replication after viral entry, indicating the alteration of the inhibition mechanisms of curcumin against virus infection via RH 40/Tween 80 micelle formulation. Furthermore, a solid formulation (Cur-SM) of Cur-M was successfully developed by a one-pot physical adsorption method using a small amount of adsorbent and ~50% of curcumin/Cur-M that could be burst released from Cur-SM in 1 h, facilitating the fast-releasing applications. Ultimately, all of the results show that Cur-SM acts as a good nano-formulation of curcumin with improved solubility/dispersity in aqueous solutions and demonstrate new anti-influenza mechanisms of curcumin for pharmaceutical development.

## 1. Introduction

Viral illnesses have a major impact on public health, and some of them pose long-term health risks. The influenza virus (IAV) causes a highly contagious respiratory illness with high morbidity and mortality rates [1,2]. According to the Centers for Disease Control’s (CDC) report, influenza A (H1N1) pdm09 viruses and influenza B (Victoria) viruses caused 410,000–740,000 hospitalizations and 24,000–62,000 influenza-related deaths in the United States during the 2019–2020 season [3]. Oseltamivir (TAMIFLU^®^), a viral neuraminidase (NA) inhibitor, is the most commonly prescribed anti-IAV drug; however, problems remain, such as drug resistance, adverse drug effects, and undefined drug safety for pregnant women and fetuses [4,5]. Therefore, the development of safe, effective, and low-cost anti-IAV drugs is urgently needed.

Natural products have attracted the interest of researchers due to their low toxicities, cost effectiveness, minimal side effects, and lesser likelihoods of promoting antiviral resistance [6]. Numerous biologically active compounds, mainly polyphenolics, have been demonstrated to have antiviral activities attributed to various of mechanisms of action, including antioxidant activity, the inhibition of DNA and RNA synthesis, and the prevention of viral particle entry [7]. Curcumin, isolated from the rhizome of turmeric, is one of the most widely investigated phenolic compounds, and has been widely used for medical purposes in China and India [8]. In recent decades, many studies indicated that curcumin possesses a variety of medical properties, including anti-inflammatory [4], antioxidant [9], Alzheimer’s disease prevention [10], and anti-microbial and antiviral effects [11,12,13].

Recently, it has been demonstrated that curcumin acts as a potent anti-influenza agent that exerts its anti-viral effect through multiple mechanisms. For example, curcumin prevents viral entry by inhibiting viral hemagglutinin (HA)-binding to cellular receptors and disrupting the integrity of the viral envelope [14,15,16]. However, the administration of curcumin failed to reduce the replication of the influenza A virus (IAV, strain PR8) due to its poor water solubility and low stability in neutral-to-basic environments [17,18]. Further, curcumin has a low systemic bioavailability of approximately 1%, which is a major limitation for biomedical applications [19]. Thus, it is essential to develop new strategies for curcumin delivery with efficient anti-influenza virus activity in order to unlock the true potential for curcumin as an antiviral agent.

Recently, nanotechnology for the delivery of hydrophobic active pharmaceutical components, including polymer–drug conjugates [20,21,22], polymeric micelles [23,24,25], nanoparticles [26,27], complex particles [28], and self-assemblies [29,30] have attracted much attention. Among these techniques, encapsulation-based micelles can improve the water solubility, stability, and circulation time of hydrophobic drugs and enlarge drug loading [31]. To address the issue of poor aqueous solubility, curcumin can be encapsulated in surfactant micelles; several studies have shown that curcumin has a significantly higher solubility (~740 µg/mL) in micellar solutions [32]. Ke et al. demonstrated that interactions between surfactant micelles and curcumin are primarily driven by the interaction of the hydrophobic force of the surfactant alkyl chains with the aryl group of curcumin, as well as the interaction of the electrostatic force of the surfactant headgroup with the diketone group of curcumin [33]. Moreover, curcumin has a greater solubility and stability with neutral surfactant micelles than charged surfactant micelles, which can be attributed to curcumin’s more pronounced hydrophobic binding with neutral surfactant micelles [34]. Wang et al. reported that the binding of curcumin with surfactant micelles may be significantly influenced by both the length and unsaturation of the alkyl chains of the surfactants [34]. On the other hand, smaller-sized curcumin nanoparticles (50 nm or less) were efficiently internalized into cells and led to a greater, more significant therapeutic efficacy [35,36]. In this study, curcumin-loaded oil-free self-assembled micelles (Cur-M) with anti-influenza outcomes were first investigated, and the scalable one-pot encapsulating and solidification method for curcumin-loaded micelles (Cur-SM) that facilitates the preparation of tablets or capsules was also developed for pharmaceutical applications.

## 2. Materials and Methods

### 2.1. Materials

Curcumin (95% total curcuminoid content) was purchased from Alfa Aesar (Haverhill, MA, USA). Kolliphor RH40, Tween 80, and Neusilin US2 were purchased from BASF (Ludwigshafen, Germany), Sigma-Aldrich (St. Louis, MO, USA), and FUJI (Osaka, Japan), respectively. Other chemicals were purchased from Sigma-Aldrich. Cells used in this study were purchased from the American Type Culture Collection (ATCC), including Madin-Darby canine kidney (MDCK) cells (ATCC, ATCCPTA-7909), human A549 cells (ATCC, CCL-185™), and human embryonic kidney (HEK293T) cells (ATCC, CRL-3216™). Madin-Darby canine kidney (MDCK), human embryonic kidney 293T, and human adenocarcinoma lung A549 cells were cultured in Dulbecco’s modified Eagle’s medium (DMEM, HyClone, Logan, UT, USA) supplemented with 5% or 10% fetal bovine serum (FBS), 0.1 mM non-essential amino acids, 1.0 mM sodium pyruvate, 0.22% sodium bicarbonate, and antibiotics (penicillin-streptomycin (Gibco BRL, Life Technologies Corp., Carlsbad, CA, USA) at 37 °C in a humidified atmosphere of 95% air and 5% CO_2_. IAV strain PR8 (A/Puerto Rico/8/1934, H1N1) was used (kindly provided by Dr. Laurence Tiley, Department of Veterinary Medicine, University of Cambridge, UK) in this study. Virus stocks were propagated in MDCK cells, and the titer was measured by a standard plaque assay.

### 2.2. Preparation of Cur-M

Micelle formulations were prepared by the self-assembly technique, which was combined and modified by self-emulsifying systems [37]. In general, surfactant and co-surfactant were dissolved in the solvent (ethanol), and then curcumin was added to the mixture with slight stirring at 60 °C. After removing the solvent by using a rotary evaporator, the self-assembly formulations were easily prepared. An appropriate amount of water was used for the hydration to achieve the desired concentration prior to use. Briefly, formulations 1 and 2 consisted of 100 mg or 500 mg of polyethylene glycol (PEG 400) as a surfactant, respectively. Curcumin was added while PEG 400 was completely dissolved in ethanol, then the solution was gently stirred at 60 °C. Finally, a rotary evaporator was applied for solvent removal to generate formulations 1 and 2. In a similar process, kolliphor RH 40 (RH 40) and propylene glycol (PG) were used separately as co-surfactants, with PEG 400 as a surfactant to generate formulations 3 and 4. For formulation 5, 10 mg of Tween 80 was used in the water phase as a stabilizer for hydration with PEG 400/RH 40 as the surfactant/co-surfactant. For formulations 6 and 7, RH 40 was used as a surfactant and Tween 80 or PEG 400 were utilized as co-surfactants, respectively. Finally, RH 40/Tween 80 was used as a surfactant/co-surfactant for Micelle (without curcumin) and Cur-M (with curcumin) formulations. The prepared Cur-M was stored at room temperature and kept in the dark for further evaluation.

### 2.3. Characterization of Particle Size and Morphology of Cur-M

The mean particle diameters and size distributions of the micelles and Cur-M were determined by the dynamic light scattering (DLS, Malvern Nano-ZS) technique. The micelle and Cur-M samples (10 mg each) were dissolved in 2 mL of water at 25 °C, and the data are reported as the mean values of triplicate measurements.

Images of the micelles and Cur-M were observed using transmission electron microscopy (TEM), in which 10 mg samples were dissolved in 50 mL of water and negative stained with 0.5% phosphotungstic acid (PTA). The shapes of the micelles and Cur-M were observed under an atomic force microscope (AFM), in which 10 mg samples were dissolved in 2 mL of water and then 20 μL of the sample solution was dropped onto a silicon chip.

### 2.4. Drug-Loading Rate and Entrapment Efficiency Measurements of Cur-M

The determination of curcumin loading rates and entrapment efficiencies in micelles analyzed by high performance liquid chromatography (HPLC) were carried out as previously reported procedures with slight modifications [38,39]. Briefly, curcumin was determined by a Hitachi Chromaster L2455 diode array detector equipped with a Hitachi Chromaster L2200 pump and a Luna 5 μm C18 column (4.6 × 250 mm). An isocratic system of two solvents, i.e., 45% acetic acid (2%) solution (solvent A) and 55% acetonitrile (solvent B), was used as the mobile phase. The injection volume was 20 μL, and the flow rate was 1 mL/min. Detection was performed using a diode array detector with a wavelength of 425 nm. Curcumin-loaded micelle dispersions were filtered using 0.45 μm filters to separate unentrapped curcumin from the Cur-M. The filtrates were diluted with methanol to dissolve micelle and were determined by HPLC. The drug-loading rate and entrapment efficiency were calculated by using Equations (1) and (2), respectively.

(1) $$DL\%=\frac{Amount\;of\;entrapped\;curcumin}{Total\;amount\;of\;micelle}\times 100\%$$

(2) $$\mathrm{EE}\%=\frac{Amount\;of\;entrapped\;curcumin}{Total\;amount\;of\;curcumin\;initial\;used}\times 100\%$$

### 2.5. In Vitro Cytotoxicity

MDCK, 293T, and A549 cells were seeded 24 h prior to treatment and allowed to reach 80% confluency. Cell culture media were replaced for treatment with fresh medium containing 60, 30, or 15 µM curcumin, Cur-M, DMSO (as solvent control), or mock treatment (DMEM only). After 12 h of treatment, the cell viability was measured based on the total cell count and trypan blue staining.

### 2.6. Virus Infection and Plaque Assay

For virus infection, MDCK cells at 6 × 10^4^ cells/well were seeded in a 24-well plate (Costar^®^, Corning, AZ, USA) one night prior to virus infection. Cell monolayers were washed twice with PBS buffer and then infected with IAV strain PR8 (either 2000 or 100 plaque formation units, pfu). After 1 h of incubation, the viral inoculum was replaced with a fresh culture medium (DMEM) containing 1 mg/mL TPCK-trypsin and cultured to the designated time (e.g., 24 h). The viral yield, supernatants containing viral progenies, were harvested at 24 h post-infection (hpi), and the viral progeny titer was determined by a standard plaque assay.

A plaque assay was routinely conducted to measure the viral titer. Briefly, confluent MDCK cell monolayers in 12-well culture plates (Costar) were infected with 10-fold serially diluted IAV. After 1 h of incubation, the viral inoculum was replaced with serum-free DMEM containing 1 mg/mL TPCK-trypsin and 0.6% agarose. After 48 h of incubation, the infected cells were fixed with 100% methanol for 30 min. Subsequently, the agarose overlays were removed. The cell monolayer was then stained with crystal violet for 2 h to visualize the plaques.

### 2.7. Time-of-Drug-Addition Assays during Virus Infection

A time-of-drug-addition assay was employed to explore the likely mechanism of the tested compounds (curcumin and Cur-M) and was compared with controls which included void polymer (carrier-only control), DMSO (solvent control), or culture medium only (mock control). The compounds were tested on cells at three distinct stages; (I) Pre-treatment: the compound chosen for treatment was given prophylactically to the cells at 16 h prior to infection, while being absent in the course of the infection; (II) Co-treatment: the compound was mixed with the virus inoculum and incubated simultaneously with the cells at the stage of virus-cell attachment, and the culture medium was replaced after 1 h of incubation; and (III) Post-entry treatment: the compound was added to the cells 1 h post-virus infection and remained in the culture medium during infection.

### 2.8. Preparation of Curcumin-Loaded Solid Self-Assembly Micelles (Cur-SM)

A solid formulation of curcumin self-assembly micelles (Cur-SM) was prepared. The kolliphor RH 40 (140 mg) and Tween 80 (10 mg) were dissolved in 200 mL of ethanol, followed by the addition of curcumin (10 mg) with stirring at 60 °C. An adsorbent of neusilin US2 (80 mg) was added to the Cur-M with stirring and the rotary evaporator to remove the solvent until the solid Cur-SM formulation appeared. The prepared Cur-SMs were stored at room temperature and kept in dark for further evaluation.

### 2.9. Water Solubility/Dispersity Study of Cur-M and Cur-SM

Curcumin, Cur-M, or Cur-SM (each containing curcumin 50 mg) was added to 1.5 mL of water. The solution was vortexed for 5 min, put in a dry bath at 50 °C for 6 h, and centrifuged at 10,000× *g* rpm for 10 min. Supernatants were collected and mixed with methanol (1:1, *v*/*v*) to stabilize the curcumin in water. The mixture (200 μL) was diluted with methanol, and then the presence of curcumin was determined by high-performance liquid chromatography (HPLC).

### 2.10. Stability of Self-Assembled Curcumin-Loaded Micelles in Different pH Buffers

Curcumin, Cur-M, and Cur-SM at different pH conditions were prepared using HCl solution (pH 1.2) and phosphate buffers (pH 6.8 and pH 7.4). Stock curcumin solution was prepared by adding 6 mg curcumin into 1 mL methanol. The Cur-M and Cur-SM stock solutions were prepared, respectively, by adding Cur-M and Cur-SM into 1 mL water with the equivalent amount of curcumin 6 mg. A total of 100 μL of each stock solution was suspended in 15 mL different pH solutions prepared using HCl solution (pH 1.2) and phosphate buffers (pH 6.8 and pH 7.4). Each sample was placed in a shaker at 37 °C before 100 μL of the supernatant was removed and mixed with 900 μL of methanol at different time intervals (0, 0.5, 1, 1.5, 3, 6, and 12 h). After diluting, the amount of curcumin in the mixture of methanol solution was determined by HPLC.

### 2.11. In Vitro Release Profile of Self-Assembled Curcumin-Loaded Micelles

The amount of free or formulated curcumin released from Cur-M and Cur-SM was studied as following the procedure reported by Khalil et al. [40]. Briefly, Cur-M and Cur-SM (containing 0.2 mg of curcumin) were suspended in 1 mL of PBS (0.01 M, pH 7.4), and the resulting suspension was placed in an Eppendorf tube. The tubes were kept in a shaker (100 rpm) at 37 °C. At the sampling time, each tube was centrifuged (10,000 rpm) for 5 min, and the supernatant was collected and diluted with methanol to disrupt the micelle and dissolve the curcumin. The residual pellet after centrifugation was resuspended with fresh PBS and incubated until the next sampling time. Finally, 20 μL of the diluted sample in methanol was injected into HPLC to determine the amount of curcumin released at different time intervals.

### 2.12. Statistical Analysis

All of the experimental data were analyzed using one-way ANOVA along with Tukey’s post hoc test. * *p* < 0.05, ** *p* < 0.01, *** *p* < 0.001 were considered significant.

## 3. Results and Discussions

### 3.1. The Cur-M Emulsifying Capability by Different Surfactants and Co-Surfactants

**Figure 1:**
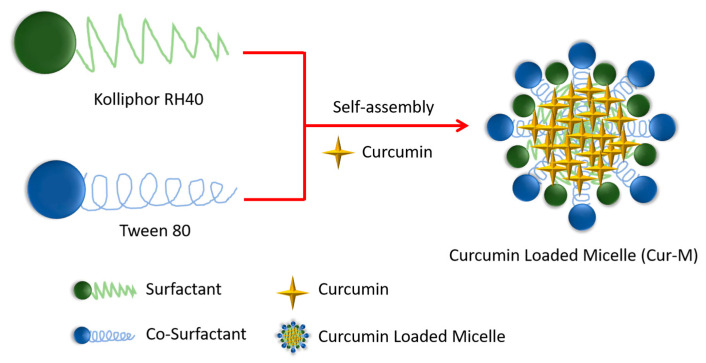
Schematic illustration for the preparation of the Cur-M.

The one-pot processes of the curcumin-loaded self-assembled micelles were successfully prepared using the various surfactants and co-surfactants. The compositions are shown in Table 1. Polyethylene glycol (PEG) has been utilized as a surfactant in the formation of polymeric micelles to improve stability and solubility of curcumin [41,42]. However, increasing the amount of PEG-400 by up to 50-fold was incapable of curcumin-loaded micelles formation in the desired size range (Table 1, formulations one and two). Hence, either Kolliphor RH 40 (RH 40) or propylene glycol (PG) was used as a co-surfactant in order to stabilize or reduce the curcumin-loaded micelles particle sizes. The results show that RH 40 effectively reduces the particle size of curcumin-loaded micelles to 115 nm while PG increases the particle size to 322 nm (Table 1—formulations three and four). Previous studies reported that the critical micelle concentration (CMC) of RH 40 was only 0.02%, which could easily form nanoparticles but lacked long-term stability for curcumin-loaded micelles [43]. Therefore, Tween 80 was applied in the water phase to stabilized the curcumin-loaded micelles, and the results show that Tween 80 effectively reduces the particle size to approximately 13 nm (Table 1, formulation five). Tween 80 and PEG were also tested in this curcumin-loaded micelle system to see which was more effective at reducing particle size, and the results show that Tween 80 is more effective in this regard (Table 1, formulations six and seven). Notably, RH 40/Tween 80 led to the formation of a more stable Cur-M with a particle size in the order of 13 nm under the water phase condition, as shown in Figure 1. Thus, this suggests that the other series of surfactants are not suitable for producing micelles of smaller sizes. Remarkably, the curcumin-loading rate was increased to 6.1% and entrapment efficiency to 97.9%. These results indicate that Cur-M is formed by the micellar self-assembly process via the encapsulation of curcumin in RH 40/Tween 80 (at a 14:1 ratio).

### 3.2. Characterization of Cur-M

**Figure 2:**
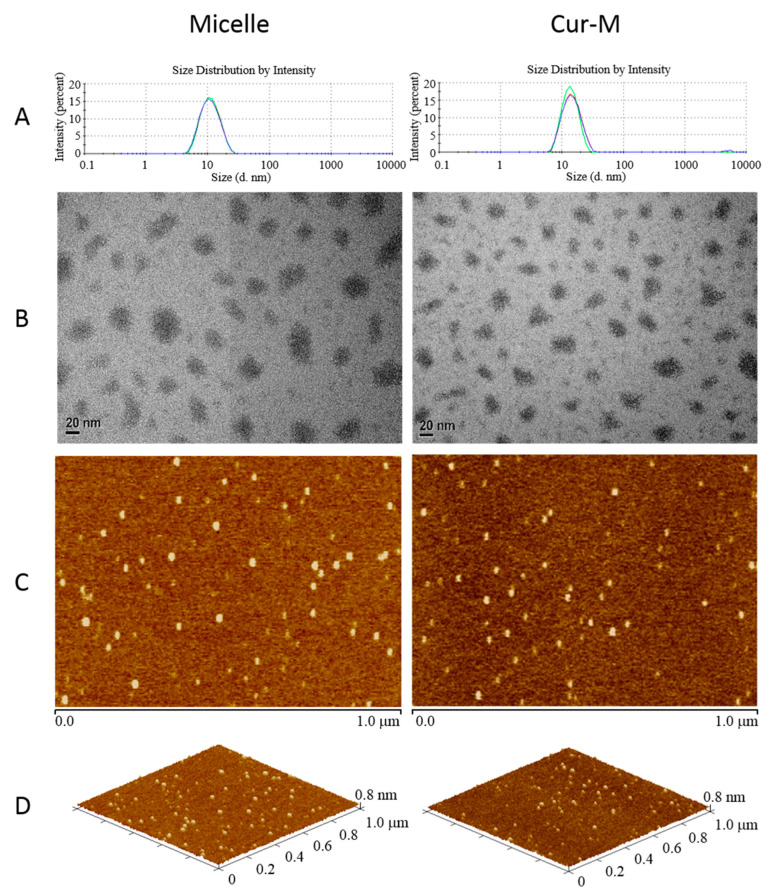
Size distributions and morphologies of void micelles and Cur-M. (**A**) Particle size distribution was determined by dissolving 10 mg samples in 2 mL of double distilled water (ddH_2_O) and was analyzed by dynamic light scattering (DLS) at 25 °C. (**B**) TEM images were acquired by dissolving 10 mg samples in 50 mL of ddH_2_O and negative staining with 0.5% phosphotungstic acid (PTA). AFM (**C**) phase images and (**D**) 3D images were acquired by dissolving 10 mg samples in 2 mL of water and then dropping 20 μL of the sample solution onto a silicon chip.

The sizes of Cur-M and curcumin-free micelles were analyzed by the dynamic light scattering (DLS) method. As shown in Figure 2A, the micelles without curcumin and with curcumin showed small average diameters of 10.27 ± 0.109 nm and 13.55 ± 0.208 nm along with narrow PDIs of 0.110 ± 0.004 and 0.144 ± 0.027, respectively (Table 1). The traditional self-nano emulsifying drug delivery system (SNEDDS) was composed of an oil phase, a surfactant and water [44]. In the present study, the micelles self-assembly system was slightly modified by surfactant and co-surfactant in the water phase. Even in the absence of the oil phase, curcumin was still observable in the encapsulated micellar form. Further, transmission electron microscopy (TEM) images demonstrated the presence of Cur-M of 10–30 nm (Figure 2B), which was compatible with the DLS data. Additionally, Cur-M particle locations could be easily confirmed by atomic force microscopy (AFM) phase images (Figure 2C). The utilization of 3D AFM images further confirmed the morphology, and the results verified that both the void micelles and Cur-M were spherical in shape (Figure 2D). These results indicate the formation of uniformly sized and stable Cur-M.

### 3.3. In Vitro Cytotoxicity and Anti-Influenza Activity of Cur-M

**Figure 3:**
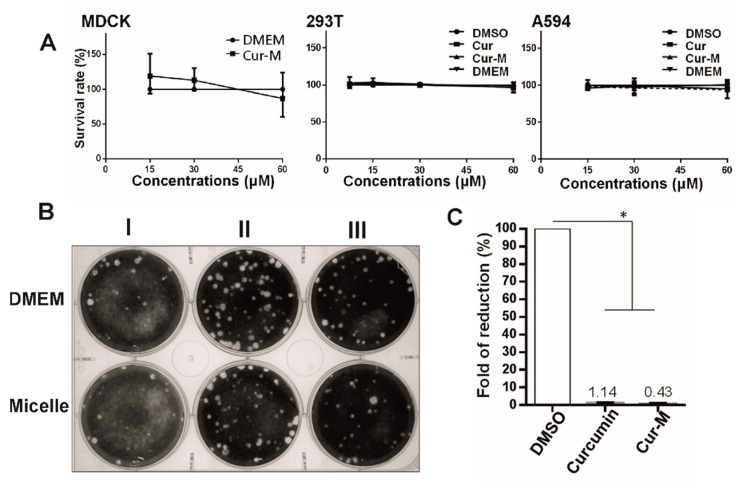
(**A**) Cytotoxicity of curcumin and Cur-M. Cytotoxicity was estimated based on the cell survival rate. In brief, three cell lines (Madin–Darby canine kidney (MDCK), 293T, and A549) commonly used for influenza propagation were treated with various concentrations of curcumin, Cur-M, and DMSO for 24 h. Cell survival rate was monitored by total vital cells counts in each treated group relative to that of mock control sample. Results from three independent experiments were plotted. (**B**) The effect of the micelle on the plaque-forming ability of the influenza virus was initially determined by a time-of-drug-addition assay, in which the tested compounds were added to the culture medium at 16 h prior to infection (pre-treatment, I), at the time of virus infection (co-treatment, II), or after viral adsorption (post-entry treatment, III). (**C**) The effect of curcumin and Cur-M on viral production. MDCK cells were continuously treated with DMSO, 30 μM curcumin, or Cur-M during influenza A virus (IAV, 2000 pfu) infection. At 24 h post-infection (hpi), the yield of viral progenies was determined. The yields relative to the mock treatment from three independent experiments were plotted. Statistically significant differences compared with the DMSO group are indicated by * (*p* < 0.05).

The cytotoxic effects of the Cur-M and native curcumin were examined on the MDCK, 293T, and A549 cells. As shown in Figure 3A, cell viability was obtained by the MTT assay performed 24 h after treatment with native curcumin, Cur-M, or DMSO as the control. Compared with the control group, native curcumin and Cur-M (60 μM) showed no significant cytotoxic effects. Moreover, our previous experiments on MDCK cells showed a lack of significant cytotoxicity in the DMEM control and curcumin of up to 60 μM [16]. The self-assembled void micelles were evaluated for anti-influenza activity by a time-of-addition assay to exclude possible anti-viral effects resulting from the carrier itself. As indicated in Figure 3B, the plaque-formation ability of the influenza virus was not affected by the micelle (curcumin-free micelle) when compared with the control group of DMEM culture medium, irrespective of the stage at which the micelle was introduced. Our previous report showed that curcumin could suppress the influenza A virus (IAV, strain PR8) [9]. The anti-viral effects on MDCK cells of curcumin (30 μM), Cur-M (30 μM), and the solvent control (DMSO) prior to and throughout IAV infection (2000 plaque formation units (pfu)) are displayed in Figure 3C; treatment with curcumin or Cur-M remarkably reduced influenza propagation to approximately 1% of that in the DMSO control group. These observations suggested that Cur-M is much more potent than native curcumin against IAV.

### 3.4. Investigation of the Anti-Influenza Efficacy of the Cur-M

**Figure 4:**
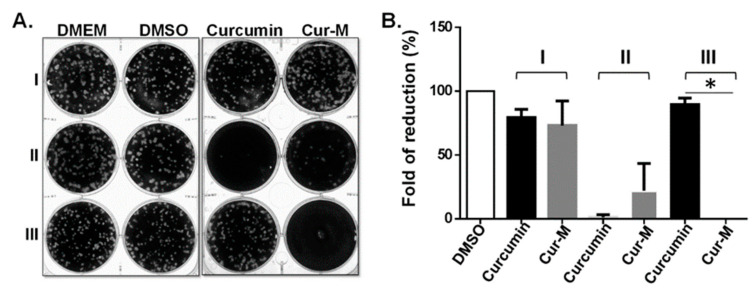
Curcumin-loaded self-assembled micelles (Cur-M) exerted potent, but distinct, anti-influenza activity. MDCK cells were treated with Dulbecco’s modified eagle’s medium (DMEM, mock control), DMSO (solvent control), curcumin, or Cur-M at various times of influenza A virus (IAV) infection (100 pfu), including pre-treatment (I), co-treatment (II), or post-entry treatment (III). The effect on the plaque-forming ability (infectivity) is shown (**A**), and the results from three independent experiments are plotted (**B**). Statistically significant differences compared with the native curcumin group are indicated by * (*p* < 0.05).

To examine the anti-viral effect of Cur-M, the infectivity of IAV was assessed by a time-of-drug-addition assay, in which the plaque-forming ability was measured using the compounds at different time intervals, i.e., before (pre-treatment), during (co-treatment), and after (post-entry treatment) virus infection (Figure 4A). The assay corresponds to the possible modes of action, such as targeting at the cellular level, stage of virus-cell attachment, and viral replication after entry, respectively. In this system, the plaque number indicates the remaining infectivity after treatment. The results show that pre-treatment with neither of the compounds (curcumin or Cur-M) leads to any significant anti-viral effect in comparison to the DMEM and DMSO control groups. In contrast, a significant reduction in IAV infectivity was observed for both curcumin and Cur-M when treatment was delivered at the time of virus attachment (co-treatment); however, Cur-M was less effective than the native curcumin in this co-treatment assay. Intriguingly, post-entry treatment with 30 µM Cur-M completely abrogated the viral infection, whereas treatment with curcumin failed to reduce the replication of IAV (Figure 4A,B). These findings indicate that Cur-M cannot effectively block HA activity by intermolecular interaction to prevent viral entry at the initial infection stage. Therefore, the micelle formulation of curcumin increases the overall cellular bioavailability and enhances stability and intracellular concentration. These results suggest that Cur-M uptaken by cells may exert a direct or indirect interaction with the IAV replication cycle after the viral entry stage in the post-entry treatment stage.

The anti-viral application of curcumin may not be limited to the common flu, but could also combat other viral infections, especially for severe viral infections lacking effective treatment, such as the COVID-19 infection caused by the severe acute respiratory syndrome corona virus 2 (SARS-CoV-2) responsible for the current global pandemic. The possible anti-viral effect of curcumin has been tested on the COVID-19 infection [45]. It has previously been reported that an oral curcumin nano-formulation can significantly improve recovery time in COVID-19 hospitalized patients [46]. In our study as well, curcumin-loaded solid self-assembled micelles effectively suppressed IAV replication in the infected phase, and research into an orally administered curcumin-loaded solid self-assembled micelles as a potential treatment of COVID-19 infection in the early and infected phase is ongoing in our lab.

### 3.5. Preparation of Cur-SM and Morphological Changes

**Figure 5:**
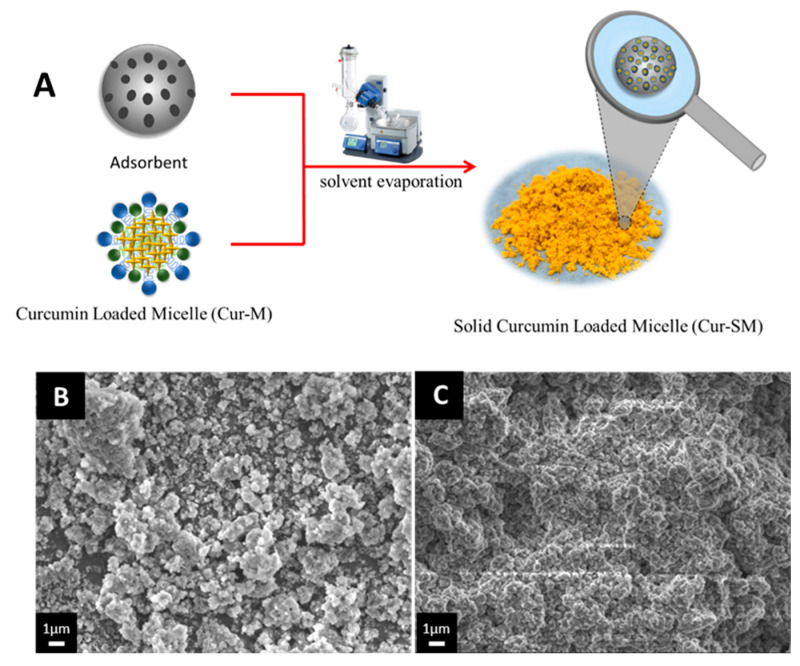
(**A**) Schematic illustration of preparation of the Cur-SM. (**B**) Scanning electron microscopy (SEM) images of the adsorbent (Neusilin US2) and (**C**) curcumin-loaded self-assembled micelles (Cur-SM). Images were acquired by placing powder samples on a graphite surface and coated with gold by an ion-sputter coater.

The Cur-SM was prepared by the solvent evaporation method, as shown in Figure 5A. The Neusilin US2 and Cur-M solutions were physically mixed and dried under vacuum. The solid formulation of curcumin (Cur-SM) was prepared by mixing Cur-M and Neusilin US2. The solid formulation of Cur-SM is more preservable, with an easier processing step for pharmaceutical application. The surface morphologies of the pure Neusilin US2 and Cur-SM were examined by scanning electron microscopy (SEM) images. The SEM image of pure Neusilin US2 showed a rough surface and a broad size distribution (Figure 5B). Additionally, when Cur-M adsorbed to Neusilin US2, the surface granularity and roughness were still visible (Figure 5C). These findings suggest that Cur-M may be partially deposited on Neusilin US2’s surface while still being absorbed into Neusilin’s porous structures. Similar outcomes for Cremophor EL adsorbed on Neusilin US2 were reported, with only partial characteristics lost while still achieving satisfactory tableting performance [47].

### 3.6. Water Solubility/Dispersity Study of Cur-M and Cur-SM

**Figure 6:**
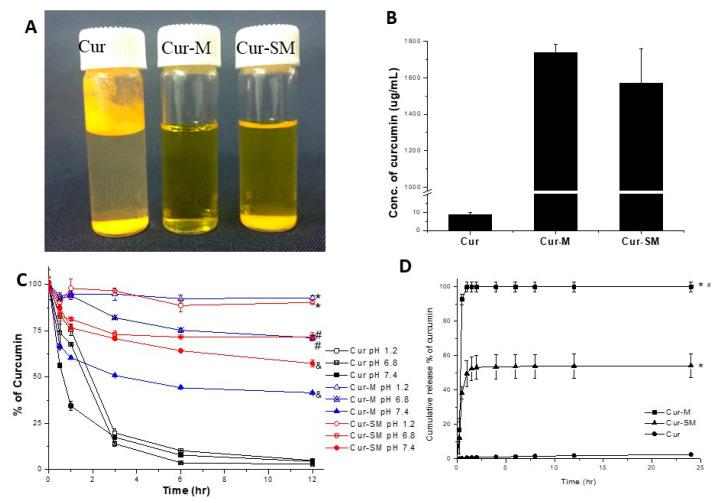
(**A**) The visual appearances of curcumin and curcumin-loaded micelles. (**B**) Aqueous solubility/dispersity. (**C**) pH stability of formulated curcumin in HCl solution (pH 1.2), phosphate buffer (pH 6.8), and phosphate buffer (pH 7.4). Statistically significant differences (*p* < 0.05) are indicated by * using native curcumin at pH 1.2, # native curcumin at pH 6.8, and & native curcumin group at pH 7.4 for comparison. (**D**) In vitro release profile of formulated curcumin. Samples were suspended in PBS at pH 7.4 and shaken at 37 °C. Cur: native curcumin; Cur-M: curcumin-loaded self-assembled micelle; and Cur-SM: curcumin-loaded solid self-assembled micelle. Data are expressed as mean ± SD values (*n* = 3). Statistically significant differences are indicated by * (*p* < 0.05).

The solubilities/dispersibilities of Cur-M and Cur-SM (curcumin 50 mg/1.5 mL) in water was compared with the native curcumin. Most of the curcumin floated on water or precipitated in the bottom (Figure 6A). In contrast, Cur-M and Cur-SM significantly dispersed in water. The solution was clear with a slightly yellow color, but the curcumin solution was colorless owing to its poor solubility. Additionally, Cur-M was dispersed in water as a nanosuspension, whereas Cur-SM showed slight turbidity and some precipitation because of the insoluble adsorbent (Figure 6A). Moreover, the solutions were subjected to HPLC analysis to confirm the presence and exact soluble/dispersed concentrations of curcumin in water. The results show that the solubility of native curcumin was 9 μg/mL. In comparison, the curcumin solubility/dispersity of the Cur-M and Cur-SM were 193-fold (1737 μg/mL) and 174-fold (1571 μg/mL) higher than that of native curcumin (Figure 6B), respectively. Therefore, the solubility/dispersity of the formulations in increasing order was native curcumin < Cur-SM < Cur-M.

### 3.7. Stability of Formulated Curcumin in Different pH Conditions

Curcumin instability in the physiological environment is a major obstacle for biomedical applications. To assess the curcumin stability at various pH conditions, native curcumin, Cur-M, and Cur-SM were incubated in different pH ranges using HCl (pH 1.2) and phosphate buffers (pH 6.8 and pH 7.4) (Figure 6C). It appeared that the native curcumin quickly degraded within 3 h under all pH conditions, and the remaining quantity of curcumin was less than 5% after 12 h of incubation [48]. Furthermore, under the same conditions, the Cur-M and Cur-SM formulations were more stable than native curcumin by 19.1-fold and 18.6-fold, respectively, after 12 h incubation at pH 1.2. Finally, the results suggest that the stability increased at a lower pH. Overall, the micellar formulation of curcumin was associated with better stability in comparison with the native form.

### 3.8. In Vitro Release Profile of Curcumin from Cur-M and Cur-SM

The release profiles of Cur-M and Cur-SM as the cumulative release portion of curcumin was carried out under the physiological condition of pH 7.4 (Figure 6D). Obviously, the measured curcumin, including free curcumin or formulated curcumin, from the Cur-M samples reached 93% of the original 0.2 mg curcumin in the supernatant, whereas only 2.5% of curcumin can be detected in the supernatant in the curcumin control group for up to 24 h due to the hydrophobic nature of curcumin, indicating the improved water dispersion property of curcumin using micelle encapsulation. A low curcumin concentration (5%) in an aqueous environment of unformulated curcumin using a comparable procedure has been previously reported [37]. Notably, the detected curcumin from Cur-SM significantly reached ~50% (balanced max. concentration) within 1 hr. According to Clint’s micellization theory, the ideal critical micelle concentration (CMC) could be calculated with the molar ratio and components [49]. In this study, the anticipated CMC value of RH 40/Tween 80 micelle was 76 μg/mL, which was calculated according to the CMC value of RH40 (75.05 μg/mL) and Tween 80 (95.95 μg/mL) [50]. The concentration of RH40/Tween 80 used for curcumin encapsulation was 2800/200 μg/mL for release profile studies, higher than the CMC value. The Cur-SM in the solution gave rise to particles that measured 13.49 nm/PDI-0.047, similar to the results of the curcumin-loaded micelles. Thus, it is speculated that most measured curcumin was in the formulated form before disruption by methanol so that it could quickly release from the Cur-SM powder. The release of formulated curcumin was about 21.65-fold higher than that of unformulated curcumin for 24 h.

### 3.9. Pharmaceutical Formulations of Tablet and Capsule Formation Using Cur-SM

**Figure 7:**
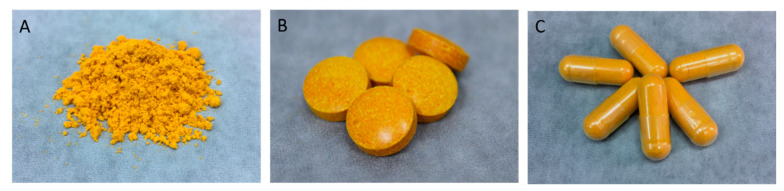
Pharmaceutical formulations of curcumin-loaded solid self-assembled micelles (Cur-SM). (**A**) Cur-SM powder, (**B**) tablet formulation of Cur-SM, and (**C**) capsule formulation of Cur-SM.

The Cur-SM were further incorporated into standard pharmaceutical formulations, such as tablets and capsules. Appropriate amounts of excipients were mixed with Cur-SM. The desktop tableting machine was used to prepare the Cur-SM tablet formulation (Figure 7B). On the other hand, a manual capsule filler was used instead to prepare the Cur-SM capsule formulation (Figure 7C). It has been noted that the preparations of tablets and capsules could provide an approach for oral drug administration against influenza virus activity. These results suggest that Cur-SM could be prepared for the large-scale manufacturing of traditional pharmaceutical formulations.

## 4. Conclusions

An optimized formulation of RH 40/Tween 80 of OFSAS was applied to encapsulate curcumin into Cur-M that more effectively suppressed IAV activity than the native curcumin when given in the post-infection stage. Remarkably, the solidification of Cur-M was successfully prepared by incorporating small amounts of the adsorbent. Compared with that of native curcumin, the water solubilities/dispersities of Cur-M and Cur-SM were increased to 1737 μg/mL (193-fold) and 1571 μg/mL (175-fold), respectively, and both existed in the micelle form under physiological conditions. Cur-SM could be easily applied to various pharmaceutical formulations, such as tablets and capsules, through a solidification process. Therefore, the Cur-SM could serve as a new anti-influenza agent with enhanced bioactivity for pharmaceutical applications

## Figures and Tables

**Table 1:** Compositions and physical characterizations of micelle formulations. Micelle: void vector of the OFSAS (oil-free self-assembly systems); Cur-M: curcumin-loaded self-assembled micelle.

	Cur (mg)	Surfactant	Co-Surfactant	Water Phase	Mean Diameter (nm)/(PDI)
1	10	PEG400 (100 mg)		-	158.5/(0.192)
2	10	PEG400 (500 mg)		-	219.1/(0.349)
3	10	PEG400 (100 mg)	RH40 (50 mg)	-	115.0/(0.297)
4	10	PEG400 (100 mg)	PG (50 mg)	-	322.6/(0.340)
5	10	PEG400 (100 mg)	RH40 (75 mg)	Tween 80 (10 mg)	13.43/(0.429)
6	50	RH40 (175 mg)	Tween80 (10 mg)	-	84.27/(0.268)
7	50	RH40 (175 mg)	PEG400 (10 mg)	-	5949/(1.0)
Micelle	0	RH40 (140 mg)	Tween80 (10 mg)	-	10.27/(0.110)
Cur-M	10	RH40 (140 mg)	Tween80 (10 mg)	-	13.55/(0.144)
Cur-SM *	10	RH40 (140 mg)	Tween80 (10 mg)		13.49/(0.047)

* Cur-SM was prepared with 80 mg adsorbent (Neusilin US2).

## Data Availability

The data presented in this study are available on request from the corresponding author.
